# Self-tuning trajectory tracking control for concrete pouring construction robots based on PID-NFTSMC and CPO algorithm

**DOI:** 10.1371/journal.pone.0324550

**Published:** 2025-05-27

**Authors:** Siwen Fan, Wanli Li, Rui Xie

**Affiliations:** School of Mechanical Engineering, Tongji University, Shanghai, China; National University of Computer and Emerging Sciences - Lahore Campus, PAKISTAN

## Abstract

This paper presented a self-tuning trajectory tracking control strategy for concrete pouring construction robots operating under external disturbances and system uncertainties. To enhance operational stability and robustness, the study integrated proportional-integral-derivative (PID) control with nonsingular fast terminal sliding mode control (NFTSMC), enabling faster convergence to the desired trajectory and reduced steady-state errors. Additionally, the study employed the crested porcupine optimizer (CPO) algorithm to automatically optimize PID control gains and NFTSMC sliding surface parameters, ensuring adaptability across varying conditions. The proposed control strategy was validated through extensive simulations, demonstrating superior trajectory tracking performance. The PID-NFTSMC controller achieved a maximum trajectory tracking error of 0.098740 and a root-mean-square (RMS) error of 0.007405 for Joint 1. For Joint 2 and Joint 3, the proposed controller exhibited maximum errors of 0.105880 and 0.088740, with RMS errors of 0.009859 and 0.007605, respectively. The convergence time for three joints was 0.1553s, 0.1540s and 0.0100s respectively. These results confirmed that concrete pouring construction robots operating had fast and high accuracy trajectory tracking and robustness against external disturbances. The findings highlight the practical significance of this approach in improving the precision and reliability of concrete pouring construction robots.

## 1. Introduction

With the increasing demands of the modern construction industry, concrete pouring construction robots are being increasingly utilized in applications such as large-scale infrastructure development, emergency rescue operations, and mining activities [1,2]. Compared to traditional manual approaches, these robots provide distinct advantages, including advanced automation capabilities, programmability, and high operational precision. Consequently, they not only enhance construction efficiency but also significantly lower labor costs and improve safety in the workplace [3,4]. In addition, concrete pouring construction robots exhibit strong adaptability to complex construction environments. By incorporating technologies such as the Internet of Things (IoT) and artificial intelligence (AI), these robots can continuously monitor construction site conditions in real time. This capability enables them to autonomously adjust operational parameters to respond effectively to dynamic external changes, thereby maintaining consistent performance under varying circumstances [5,6]. However, ensuring operational stability during concrete placement remains a critical challenge. The end effector of the robot must traverse the construction site with precise positioning, which is particularly demanding in terms of stability. One significant issue arises from the design of manipulators, which often extend tens of meters or more. To minimize weight, their structural stiffness is intentionally reduced, but this compromises their ability to resist elastic vibrations. As a result, control becomes more complex, leading to trajectory tracking errors [7]. Additionally, external disturbances and environmental uncertainties can significantly affect the reliability of the robotic system, further complicating its operation and diminishing overall performance [8].

To improve dynamic performance and enhance robustness against external disturbances and system uncertainties, methods based on the classical proportional-integral-derivative (PID) control were widely adopted in the early stages due to their straightforward design and ease of implementation [9–11]. However, controllers relying on PID faced significant limitations in handling highly nonlinear systems or intense disturbances. As a result, they struggled to adapt effectively to the complexities of construction environments. To address these challenges, sliding mode control (SMC) was developed, offering greater robustness against variations in system parameters and external disturbances in dynamic conditions [12–14]. A key feature of sliding mode control is its inherent invariance property, which enables it to maintain high accuracy in trajectory tracking even in complex and uncertain environments. For instance, Aderajew et al. proposed a self-tuning fuzzy sliding mode controller (ST-FSMC) specifically for achieving precise trajectory tracking of a 3-degree-of-freedom robotic manipulator. Simulation results demonstrated that the designed controller achieved superior tracking performance [15]. Additionally, comparative studies have utilized both classical PID and SMC controllers to evaluate trajectory and position control performance. These studies showed that SMC provided better dynamic response and improved overall system performance compared to PID controllers [16]. However, traditional SMC controllers are not without drawbacks. One prominent issue is the “chattering” phenomenon, which can accelerate wear and tear on mechanical components. Moreover, the convergence of state variables in sliding mode control is slow, which negatively impacts trajectory tracking accuracy. Another challenge lies in estimating the upper bound of the unknown nonlinear functions within the system, which complicates the implementation of the control strategy.

To address the limitations of conventional sliding mode control, researchers have developed various improved sliding mode control methods. Among these, nonsingular fast terminal sliding mode control (NFTSMC) has been recognized for its ability to achieve finite-time convergence of system states. This is accomplished by designing nonsingular terminal sliding mode surfaces, which greatly enhance both control accuracy and response speed [17–19]. Unlike traditional sliding mode control, NFTSMC eliminates the singularity problem and significantly accelerates system stability convergence. Despite these benefits, its adaptability to nonlinear disturbances and complex dynamic models remains an area requiring further development. Additionally, integral sliding mode control has been introduced to enhance the system's ability to respond quickly to faults and external disturbances. By incorporating an integral term into the sliding mode surface, this approach effectively reduces steady-state errors caused by disturbances. This characteristic makes it particularly well-suited for systems that must operate stably over extended periods [20–22]. However, its performance is constrained when dealing with systems exhibiting strong coupling or highly complex dynamics. To mitigate the chattering phenomenon inherent in traditional sliding mode control, higher-order sliding mode control has been proposed. By constructing higher-order sliding mode surfaces, such as second- or third-order derivative surfaces, this method significantly reduces the amplitude of chattering during switching [23–25]. As a result, it is especially suitable for applications requiring high-precision control. Nevertheless, the implementation of higher-order sliding mode control is more complex, particularly in real-time scenarios where computational resources are limited, posing challenges for practical applications.

Although the aforementioned improvement techniques address some of the limitations of conventional sliding mode controllers (SMCs), practical applications often require a control strategy that simultaneously ensures fast response, finite-time stability, and reduced chattering. To meet these demands, researchers have proposed a composite control strategy that integrates multiple methods. Backstepping, a recursive design approach, is particularly effective for managing complex system dynamics incrementally. By integrating nonsingular fast terminal sliding mode control (NFTSMC) with backstepping, the resulting control system achieves both a rapid dynamic response and finite-time stability [26–28]. Despite these advantages, this approach does not fully leverage the robustness benefits of proportional-integral-derivative (PID) controllers. It also exhibits limitations when applied to strongly nonlinear or highly coupled systems. To address these challenges, this paper proposes a control strategy that combines the strengths of PID control and NFTSMC. This integrated approach not only enhances system robustness but also achieves a fast response and ensures finite-time convergence, even in the presence of system faults and external disturbances.

Although integrating PID and NFTSMC controllers enables concrete pouring construction robots to achieve strong robustness, fast response, and low trajectory tracking error, the effectiveness of this method heavily depends on selecting appropriate PID and NFTSMC controller parameters, including proportional, integral, and derivative gains. Traditionally, these parameters are determined through a trial-and-error process, which is not only time-consuming and labor-intensive but also limits the ability to achieve optimal dynamic performance. This challenge becomes particularly significant in complex environments or when dealing with strongly coupled systems, where practical engineering requirements are harder to meet. To overcome this limitation, a variety of intelligent optimization algorithms have been widely adopted in recent years for the automatic tuning of controller parameters [29–31]. These methods significantly enhance efficiency and control performance by reducing manual tuning efforts and identifying the optimal parameter combinations within the search space. For example, genetic algorithms (GA) mimic biological processes such as selection, crossover, and mutation to find optimal solutions. However, these algorithms can be slow to converge on complex problems and are prone to local optima [32,33]. Similarly, particle swarm optimization (PSO) achieves rapid approximation of global optima by modeling the social behaviors of a particle population, including position and velocity updates [34,35]. While effective for multidimensional parameter optimization, PSO is sensitive to initial parameter settings, which may affect its overall performance. Another method, the bacterial foraging algorithm (BFA), simulates the foraging behavior of bacteria to perform global searches, offering strong robustness and adaptability. However, its computational complexity increases significantly in high-dimensional search spaces [36,37]. As an advanced approach, the crested porcupine optimizer (CPO) has gained attention for its simplicity and efficiency. This algorithm simulates porcupine defense behavior to achieve convergence to the optimal solution [38,39]. Compared with other algorithms, CPO requires fewer adjustable parameters, avoiding the need for complex coordination mechanisms or extensive parameter tuning. Its low computational complexity and fast convergence make it a preferred choice for parameter optimization in various applications, offering both high efficiency and stability. The main contributions of this study are outlined as follows:

1. Compared to traditional terminal sliding mode control, nonsingular fast terminal sliding mode control (NFTSMC) eliminates the issue of singularities on the sliding mode surface. This improvement results in a more stable and computationally reliable controller design. By incorporating a nonlinear dynamic term, NFTSMC accelerates system convergence and reduces errors during the dynamic process. These advancements significantly enhance the trajectory tracking precision and reliability of concrete pouring construction robots.
2. The combination of PID control with NFTSMC effectively mitigates the chattering problem associated with sliding mode control. Additionally, it improves the system's responsiveness and reduces the time required to approach the sliding mode surface, further enhancing control performance.
3. The use of the crested porcupine optimizer (CPO) for automatic adjustment of PID parameters enhances the system's robustness and its ability to reject disturbances. This optimization ensures a fast response to uncertainties and external disturbances, improving the resilience and accuracy of concrete pouring construction robots in tracking the desired trajectory.

## 2. Concrete pouring construction robots

### 2.1. Physical prototype description

**Fig 1:**
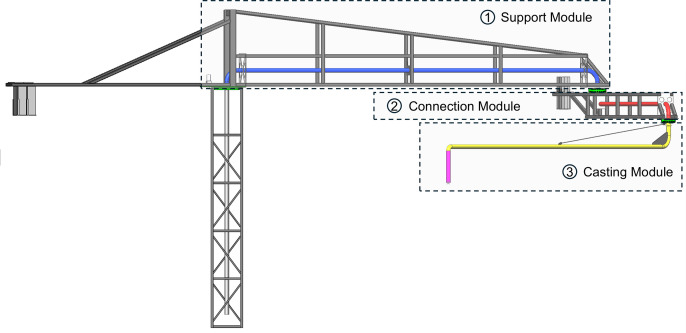
Mechanical structure of concrete pouring construction robots.

As illustrated in Fig 1, this paper introduces a concrete pouring construction robot built around a three-degree-of-freedom robotic manipulator. The robot features a tower-based structure as its primary support, which can be flexibly mounted on a mobile casting platform to adapt to various construction scenarios. Unlike traditional concrete pump trucks commonly used in casting operations, this robot incorporates an independent base design. This design significantly enhances its wind resistance, enabling stable operation in larger construction areas.

The robot comprises three main structural components: the support module, the connection module, and the casting module. First, the support module serves as the load-bearing component, providing stability and ensuring the reliability of the entire robotic system. As represented by the gray-black sections in the figure, the support module adopts a truss-style standard section design reinforced with steel wire suspension cables to improve flexural rigidity. Due to the robotic arm's extended length and complex center-of-gravity distribution, the support module is further equipped with counterweights to balance mechanical forces and structural loads during operation. Next, the casting module is specifically designed to facilitate efficient concrete delivery and precise placement. This module comprises standard engineering pipelines and pipe clamps, forming a delivery system along the robotic arm's length. This system transports concrete from the supply point to the target location. At the casting end, a concrete delivery hose is attached, enabling accurate transfer of concrete to the intended area to complete the casting task. Finally, the connection module acts as the pivotal coupling mechanism linking the other structural components. Its primary role is to ensure coordination and stability among different parts of the robotic arm during operation. The connection module is designed with both load-bearing capacity and adaptability in mind, enabling the robotic arm to function effectively across varying lengths and angles while maintaining reliable performance in diverse construction environments. By integrating a modular design with an independent base structure, this concrete casting robot demonstrates enhanced wind resistance and adaptability to large-scale construction applications. The modular approach not only simplifies the design process but also provides a flexible framework for potential future functionality enhancements.

**Fig 2:**
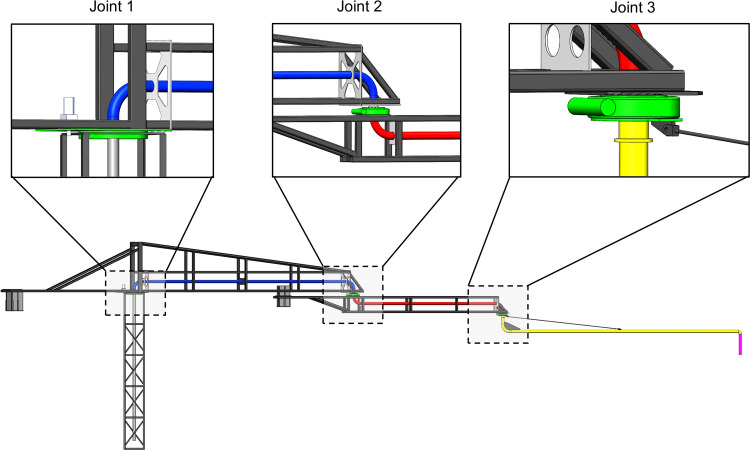
Three main joints of concrete pouring construction robot.

As depicted in Fig 2, the concrete pouring construction robot is equipped with three rotational joints, each utilizing standard engineering-grade slewing bearings. These bearings are used to drive and control each section of the robotic system. Each rotational joint is powered by a corresponding servo motor, which provides precise actuation and accurate control for its associated section. The configuration of these three rotational joints grants the robot three degrees of freedom, allowing for redundant motion within the x-y plane. This redundancy enhances the robot's flexibility in selecting casting paths, thereby simplifying the structural design of the system. Such a design is particularly advantageous in construction environments with high levels of complexity, as it increases the operational adaptability of the robot and improves structural stability under extreme working conditions. Moreover, this design contributes to enhanced construction efficiency and reduced operational costs. Compared to traditional concrete pump trucks, the robot's reduced degrees of freedom enable the implementation of intelligent control algorithms with greater ease. This not only improves computational efficiency but also simplifies system integration, making the robot more practical for diverse construction scenarios.

**Fig 3:**
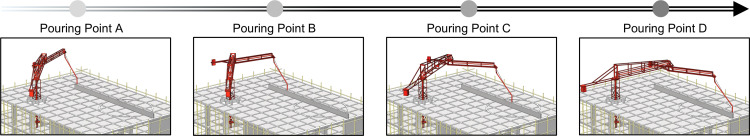
Schematic of a concrete pouring construction robot operating in a straight line.

Fig 3 illustrates the application of the concrete pouring construction robot in slab casting for high-rise buildings. Positions A, B, C, and D represent intermediate control points along the pouring path. By coordinating the movement of its three rotational joints, the robot accurately positions the concrete delivery hose at the designated pouring locations, enabling precise execution of the slab casting task. The example shown in the figure demonstrates a straight-line pouring trajectory. The robot follows the pre-defined path, passing through each intermediate point and delivering concrete to the target areas. By utilizing the flexibility provided by its rotational joints and its ability to achieve precise positioning, the robot ensures accurate path tracking. This capability allows it to adapt effectively to the complex construction conditions typically encountered in high-rise building projects.

### 2.2. Kinematics

**Fig 4:**
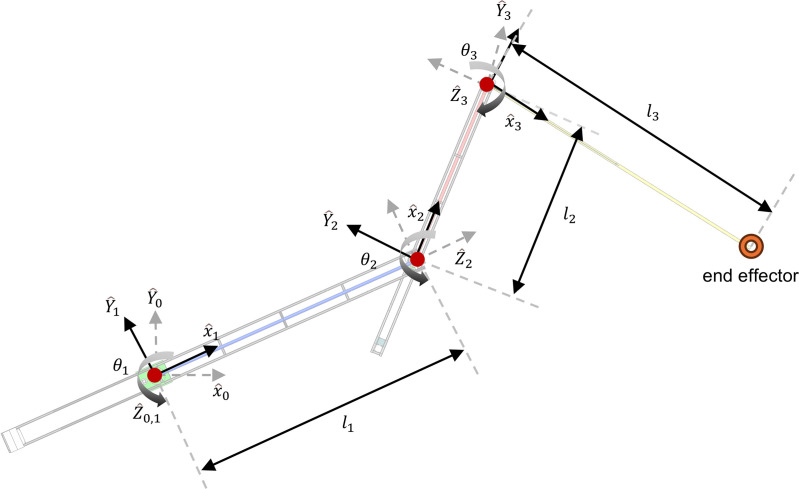
General model of manipulator of concrete pouring construction robot.


Considering that the concrete pouring construction robot operates in Cartesian space while its motion control is performed in joint space, it is essential to establish a kinematic model to transform coordinates from Cartesian space to joint space. Based on the robot's mechanical structure and the Denavit-Hartenberg (D-H) convention, a kinematic model has been developed to address this requirement. As illustrated in Fig 4, the robot features four coordinate systems: the base coordinate system and three joint coordinate systems. To simplify the implementation of the algorithm, the base coordinate system is fixed at the position of the first rotational joint, as the base height varies dynamically depending on the specific engineering application. Coordinate transformations are defined between each of the coordinate systems, ensuring accurate mapping of motion. Furthermore, the end effector possesses an independent translational motion relative to joint 3, adding flexibility to its operation. According to the D-H convention, the six degrees of freedom defining the robot's pose are reduced to four parameters, as represented in the following equations:

$$ii-1T=Rot(z_{i-1},\theta_{i}timesTrans(z_{i-1},d_{i})\times Trans(x_{i-1},a_{i-1})\times Rot(x_{i-1},\alpha_{i-1})$$ (1)

where $\theta_{i}$ denotes the rotational angle at joint *i* (Joint Angle) and *i* = 1,2,3 for the concrete pouring construction robot. $\alpha_{i}$ denotes the twist angle between links (Twist Angle). Since the robot's z-axis is fixed and vertical, all $\alpha_{i}=0$. $a_{i}$ denotes the link length between joints (Link Length) and $d_{i}$ is the link offset (Link Offset). Eq (1) can be expanded as follows:


$$ii-1T=\left[\begin{array}{cccc} \text{cos}\theta_{i} & -\text{sin}\theta_{i}\cos\alpha_{i-1} & \text{sin}\theta_{i}\sin\alpha_{i-1} & a_{i-1}\text{cos}\theta_{i}\\ \text{sin}\theta_{i} & \text{cos}\theta_{i}\cos\alpha_{i-1} & \text{-cos}\theta_{i}\sin\alpha_{i-1} & a_{i-1}\text{sin}\theta_{i}\\ 0 & \sin\alpha_{i-1} & \cos\alpha_{i-1} & d_{i}\\ 0 & 0 & 0 & 1 \end{array}\right]$$ (2)

Using the forward kinematic propagation formula, the transformation from the end effector to the base coordinate system can be obtained.


$$ii-1T=\prod_{i=1}^{n}ii-1T$$ (3)

Based on the parameters in Tables 1 and 2, the transformation matrix between the end effector and the base coordinate system was derived through coordinate transformations as follows:

**Table 1:** The D-H parameter of a concrete pouring construction robot.

i	$\alpha_{i-1}$	$a_{i-1}$	$d_{i}$	$\theta_{i}$
1	0	0	$d_{1}$	$\theta_{1}$
2	0	${\text{l}}_{1}$	$d_{2}$	$\theta_{2}$
3	0	${\text{l}}_{2}$	$d_{3}$	$\theta_{3}$

**Table 2:** The relevant parameter of a concrete pouring construction robot arm.

	First	Middle	End
Link Length/m	11	7	13
Link Offset/m	0	1	1
Range of joint Angle	(−365∘,+365∘)	(−365∘,+365∘)	(−365∘,+365∘)


$$30T=10T\times 21T\times 32T\times \text{E}3T\text{=}\left[\begin{array}{cccc} n_{x} & o_{x} & a_{x} & p_{x}\\ n_{y} & o_{y} & a_{y} & p_{y}\\ n_{z} & o_{z} & a_{z} & p_{z}\\ \text{0} & \text{0} & \text{0} & \text{1} \end{array}\right]$$ (4)

Substituting the parameters of the table, the parameters of Eq (4) can be obtained:


$$\left\{ \begin{array}{cc} & n_{x}=\text{c}q_{3}\times (\text{c}q_{1}\times \text{c}q_{2}-\text{s}q_{1}\times \text{s}q_{2})-\text{s}q_{3}\times (\text{c}q_{1}\times \text{s}q_{2}+\text{c}q_{2}\times \text{s}q_{1})\\ & n_{y}=\text{c}q_{3}\times (\text{c}q_{1}\times \text{s}q_{2}+\text{c}q_{2}\times \text{s}q_{1})+\text{s}q_{3}\times (\text{c}q_{1}\times \text{c}q_{2}-\text{s}q_{1}\times \text{s}q_{2})\\ & n_{z}=0\\ & o_{x}=-\text{c}q_{3}\times (\text{c}q_{1}\times \text{s}q_{2}+\text{c}q_{2}\times \text{s}q_{1})-\text{s}q_{3}\times (\text{c}q_{1}\times \text{c}q_{2}\text{-s}q_{1}\times \text{s}q_{2})\\ & o_{y}=\text{c}q_{3}\times (\text{c}q_{1}\times \text{c}q_{2}\text{-s}q_{1}\times \text{s}q_{2})-\text{s}q_{3}\times (\text{c}q_{1}\times \text{s}q_{2}\text{+c}q_{2}\times \text{s}q_{1})\\ & o_{z}=0\\ & a_{x}=0\\ & a_{y}=0\\ & a_{z}=1\\ & p_{x}=11\times \text{c}q_{1}-13\times \text{s}q_{3}\times (\text{c}q_{1}\times \text{s}q_{2}+\text{c}q_{2}\times \text{s}q_{1})+7\times \text{c}q_{1}\times \text{c}q_{2}-7\times \text{s}q_{1}\times \text{s}q_{2}+13\times \text{c}q_{3}\times (\text{c}q_{1}\times \text{c}q_{2}-\text{s}q_{1}\times \text{s}q_{2})\\ & p_{y}=11\times \text{s}q_{1}+13\times \text{s}q_{3}\times (\text{c}q_{1}\times \text{c}q_{2}-\text{s}q_{1}\times \text{s}q_{2})+7\times \text{c}q_{1}\times \text{s}q_{2}+7\times \text{c}q_{2}\times \text{s}q_{1}+13\times \text{c}q_{3}\times (\text{c}q_{1}\times \text{s}q_{2}\text{+c}q_{2}\times \text{s}q_{1})\\ & p_{z}=2 \end{array}\right.$$ (5)

**Fig 5:**
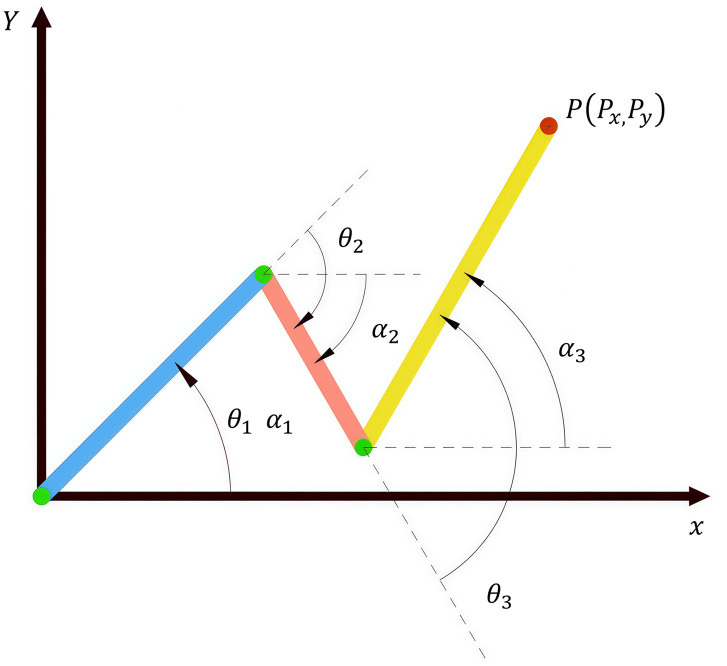
Schematic diagram of concrete pouring construction robot in a 2D coordinate system.


Based on the above-mentioned pose relationships, the inverse kinematics model of the robot can be established. As shown in Fig 4 and Fig 5, the inverse kinematics calculations are performed using the geometric analysis method, and the detailed derivation process is as follows:

$$\left\{ \begin{array}{c} {}*35lP_{x}=l_{1}\times \cos\alpha_{1}+l_{2}\times \cos\alpha_{2}+l_{3}\times \cos\alpha_{3}\\ P_{y}=l_{1}\times \sin\alpha_{1}+l_{2}\times \sin\alpha_{2}+l_{3}\times \sin\alpha_{3}\\ \alpha_{1}=\theta_{1}\\ \begin{array}{cc} & \alpha_{2}=\alpha_{1}+\theta_{2}=\theta_{1}+\theta_{2}\\ & \alpha_{3}=\alpha_{2}+\theta_{3}=\theta_{1}+\theta_{2}+\theta_{3} \end{array} \end{array}\right.$$ (6)

By solving the system of equations, the joint rotation angles corresponding to each pouring point can be determined. As shown in Fig 5, the final calculated results are presented as follows:


$$\left\{ \begin{array}{c} {}*35l\theta_{1}\text{ =}\alpha_{1}\\ \theta_{2}=P_{x}\text{-}l_{2}\arccos(\frac{{\left(P_{x}-l_{1}\cos\alpha_{1}\right)}^{2}+l_{1}l_{2}-P_{y}-l_{1}\cos^{2}\alpha_{1}}{l_{2}l_{3}\cos\alpha_{2}})-l_{1}\cos\alpha_{1}-\alpha_{1}\\ \theta_{3}=\arccos(\frac{{\left(P_{x}-l_{1}\cos\alpha_{1}\right)}^{2}+l_{1}l_{3}-P_{y}-l_{1}\cos^{2}\alpha_{1}}{l_{2}l_{3}\cos\alpha_{2}})-\alpha_{2} \end{array}\right.$$ (7)

where counterclockwise rotations are designated as positive, whereas clockwise rotations are defined as negative.

### 2.3. Dynamics model

The rotational joints of the concrete pouring construction robot were driven by servo motors, which actuated the joints based on motion signals sent by the upper-level control system. These motors rotated the three joints to the required angles, allowing the end delivery nozzle to reach the designated position. However, due to the presence of non-homogeneous concrete fluid flow within the manipulator, the pouring control system exhibited strong coupling and highly nonlinear dynamics, making it prone to environmental disturbances in complex construction scenarios. To address these challenges, a more robust controller was required to enhance the system's stability. The concrete pouring construction robot was modeled as a 3-degree-of-freedom (3DoF) rigid robot, with its dynamics describing the relationship between the servo motor torques and joint movements. The Lagrangian method was commonly used to establish the dynamic model of the robotic arm, which was represented by a second-order differential equation as follows:


$$\tau =M(q)\ddot{q}+C(q,\dot{q})\dot{q}+G(q)+\tau_{d}(q,\dot{q})$$ (8)

where τ is the control torque provided of the motor, M(q) represented the inertia matrix, $C(q,\dot{q})$ described the coupling of Coriolis and centrifugal forces, G(q) was the gravity torque matrix and $\tau_{d}(q,\dot{q})$ accounted for the effects of variations in system parameters and external disturbances. Here, $q,\dot{q},\ddot{q}$ denoted the joint angles, angular velocities, and angular accelerations of the robotic arm, respectively. Due to the presence of redundant degrees of freedom and the effects of fluid dynamics, modeling the system under this framework was highly complex. To simplify the analysis, the effects of gravity were neglected, and the dynamic equation was reduced to the following form:


$$\tau =M(q)\ddot{q}+C(q,\dot{q})\dot{q}+\tau_{d}(q,\dot{q})$$ (9)

The Eq (9) was calculated and expressed as a matrix form of the state space equations:


$$\begin{array}{c} \left[\begin{array}{cc} & \tau_{1}\\ & \tau_{2}\\ & \tau_{3} \end{array}\right]=\left[\begin{array}{ccc} M_{11} & M_{12} & M_{13}\\ M_{21} & M_{22} & M_{23}\\ M_{31} & M_{32} & M_{33} \end{array}\right]\left[\begin{array}{cc} & {\ddot{q}}_{1}\\ & {\ddot{q}}_{2}\\ & {\ddot{q}}_{3} \end{array}\right]+\left[\begin{array}{cc} & C_{1}(q,\dot{q})\\ & C_{2}(q,\dot{q})\\ & C_{3}(q,\dot{q}) \end{array}\right]\left[\begin{array}{cc} & {\dot{q}}_{1}\\ & {\dot{q}}_{2}\\ & {\dot{q}}_{3} \end{array}\right] \end{array}$$ (10)

where


$$\left\{ \begin{array}{cc} & M_{11}={\text{a}}_{1}+{\text{a}}_{2}+{\text{a}}_{4}+2{\text{a}}_{3}\cos(q_{2})+2{\text{a}}_{5}\cos(q_{2}+q_{3})+2{\text{a}}_{6}\cos(q_{3})\\ & M_{12}={\text{a}}_{2}+{\text{a}}_{4}+{\text{a}}_{3}\cos(q_{2})+{\text{a}}_{5}\cos(q_{2}+q_{3})+2{\text{a}}_{6}\cos(q_{3})\\ & M_{13}={\text{a}}_{4}+{\text{a}}_{5}\cos(q_{2}+q_{3})+2{\text{a}}_{6}\cos(q_{3})\\ & M_{21}={\text{a}}_{2}+{\text{a}}_{4}+{\text{a}}_{3}\cos(q_{2})+{\text{a}}_{5}\cos(q_{2}+q_{3})+2{\text{a}}_{6}\cos(q_{3})\\ & M_{22}={\text{a}}_{2}+{\text{a}}_{4}+2{\text{a}}_{6}\cos(q_{3})\\ & M_{23}={\text{a}}_{4}+{\text{a}}_{6}\cos(q_{3})\\ & M_{31}={\text{a}}_{4}+{\text{a}}_{5}\cos(q_{2}+q_{3})+2{\text{a}}_{6}\cos(q_{3})\\ & M_{32}={\text{a}}_{4}+{\text{a}}_{6}\cos(q_{3})\\ & M_{33}={\text{a}}_{4}\\ & C_{1}(q,\dot{q})=-{\text{a}}_{3}(2{\dot{q}}_{1}+{\dot{q}}_{2}){\dot{q}}_{2}\sin(q_{2})-{\text{a}}_{5}(2{\dot{q}}_{1}+{\dot{q}}_{2}+{\dot{q}}_{3})({\dot{q}}_{2}+{\dot{q}}_{3})\sin(q_{2}+q_{3})-{\text{a}}_{6}(2{\dot{q}}_{1}+2{\dot{q}}_{2}+{\dot{q}}_{3}){\dot{q}}_{3}\sin(q_{3})\\ & C_{2}(q,\dot{q})={\text{a}}_{3}{{\dot{q}}_{1}}^{2}\sin(q_{2})+{\text{a}}_{5}{{\dot{q}}_{1}}^{2}\sin(q_{2}+q_{3})-{\text{a}}_{6}(2{\dot{q}}_{1}+2{\dot{q}}_{2}+{\dot{q}}_{3}){\dot{q}}_{3}\sin(q_{3})\\ & C_{3}(q,\dot{q})={\text{a}}_{5}{{\dot{q}}_{1}}^{2}\sin(q_{2}+q_{3})+{\text{a}}_{6}{\left({\dot{q}}_{1}+{\dot{q}}_{2}\right)}^{2}\sin(q_{3}) \end{array}\right.$$ (11)

where ${\text{a}}_{i}$ were parameters of the robot structure and can be calculated according to the following equation:


$$\left\{ \begin{array}{cc} & {\text{a}}_{1}=0.25{\text{m}}_{\text{1}}{{\text{l}}_{\text{1}}}^{2}+{\text{J}}_{\text{1}}+({\text{m}}_{\text{2}}+{\text{m}}_{\text{3}}){{\text{l}}_{\text{1}}}^{2}\\ & {\text{a}}_{2}={\text{J}}_{2}+0.25{\text{m}}_{\text{2}}{{\text{l}}_{2}}^{2}+{\text{m}}_{\text{3}}{{\text{l}}_{\text{2}}}^{2}\\ & {\text{a}}_{3}={\text{l}}_{\text{1}}(0.5{\text{m}}_{\text{2}}{\text{l}}_{2}+{\text{m}}_{\text{3}}{\text{l}}_{2})\\ & {\text{a}}_{4}={\text{J}}_{\text{1}}+0.25{\text{m}}_{\text{3}}{{\text{l}}_{3}}^{2}\\ & {\text{a}}_{5}=0.5{\text{m}}_{\text{3}}{\text{l}}_{1}{\text{l}}_{3}\\ & {\text{a}}_{6}=0.5{\text{m}}_{\text{3}}{\text{l}}_{2}{\text{l}}_{3} \end{array}\right.$$ (12)

where the mass of each arm was calculated as a fully loaded DN125 wear-resistant concrete pipe, as shown in Table 3.

**Table 3:** The mass of each arm.

i	1	2	3
${\text{m}}_{i}/kg$	424	270	501

The rotational moment of inertia J is calculated according to the slender rod formula, and the rotary axis passes through and perpendicular to the endpoints of the rod with the following formula:


$$\left\{ \begin{array}{cc} & {\text{J}}_{1}=\frac{1}{3}{\text{m}}_{\text{1}}{{\text{l}}_{\text{1}}}^{2}\\ & {\text{J}}_{2}=\frac{1}{3}\text{(}{\text{m}}_{2}{{\text{l}}_{2}}^{2}\text{+3}{\text{m}}_{2}{{\text{l}}_{1}}^{2}\text{)}\\ & {\text{J}}_{2}=\frac{1}{3}\text{(}{\text{m}}_{3}{{\text{l}}_{3}}^{2}\text{+3}{\text{m}}_{3}{\text{(}{\text{l}}_{1}+{\text{l}}_{2})}^{2}\text{)} \end{array}\right.$$ (13)

## 3. Controller design

**Fig 6:**
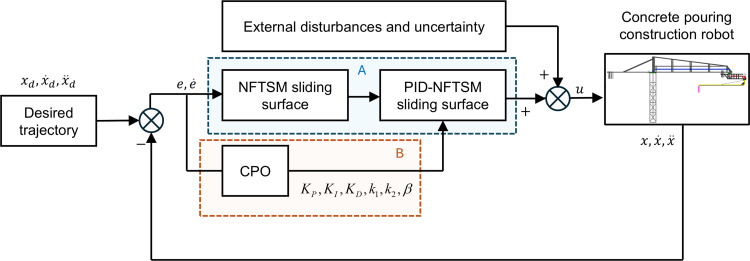
Control scheme for concrete pouring construction robots.

In actual operations, the trajectory tracking control of a concrete pouring construction robot is often influenced by external disturbances and uncertainties. To address these challenges, the proposed PID-NFTSMC method was applied to control the trajectory tracking of concrete pouring construction robots (Fig 6). In addition, a CPO optimizing algorithm was utilized to adjust the parameters of PID and NFTSMC controller automatically. The integrated control method was developed to ensure high-precision trajectory tracking performance under the extreme external disturbances and obtain fast-response. Meanwhile, the robustness was also improved.

### 3.1. Design of PID-NFTSMC control strategy

Based on Eq (9), a typical dynamic of this proposed robot manipulator can be described as follows:


$$\ddot{q}=M^{-1}(q)\left(\tau -C(q,\dot{q})\dot{q}-\tau_{d}(q,\dot{q})\right)$$ (14)

Let $x_{1}=q,$ $x_{2}=\dot{q}$ and u=τ. Thus, a state space mode based on Eq (4) can be described as follows:


$$\left\{ \begin{array}{cc} & {\dot{x}}_{1}=x_{2}\\ & {\dot{x}}_{2}=M^{-1}(x_{1})u-M^{-1}C(x_{1},x_{2})x_{2}-\Delta d\\ & y=x_{1} \end{array}\right.$$ (15)

where $\Delta d=M^{-1}\tau_{d}(x_{1},x_{2})$ represents external disturbances and uncertainty.

**Assumption 3.1:** The model of external disturbances and uncertainty is bounded by:


$$|\Delta d|\leq d_{0}$$ (16)

**Assumption 3.2:** The derivative of the external disturbances and uncertainty model is bound by:


$$|\Delta \dot{d}|\leq d_{1}$$ (17)

where $d_{0}$ and $d_{1}$ are constants. Assuming the desired trajectory is $x_{d}$. The trajectory tracking error can be described as $e=x_{1}-x_{d}$ and the desired velocity error is calculated as $\dot{e}={\dot{x}}_{1}-{\dot{x}}_{d}$. Thus, the PID sliding mode surface can be defined as follows:


$$S_{\text{PID}}=K_{P}s+K_{I}\int_{0}^{t}sdt+K_{D}\dot{s}$$ (18)

where $K_{P}$, $K_{I}$ and $K_{D}$ are the proportional, integral and derivative parameters respectively. In order to achieve fast time convergence and avoid the singular problem, a NFTSMC sliding surface is utilized as follows:


$$s=e+k_{1}e^{\left[\lambda \right]}+k_{2}{\dot{e}}^{\left[p/q\right]}$$ (19)

where $k_{1}$ and $k_{2}$ are two positive definite matrices and p and q are selected to meet the following conditions 1<p/q<2 and λ>p/q. Then, the PID-NFTSCM sliding surface can be proposed as follows:


$$S_{\text{PID}}(t)=K_{P}s(t)+K_{I}\int_{0}^{t}s(t)dt+K_{D}\frac{ds(t)}{dt}$$ (20)

Based on Eq (20), the response time of the system is highly dependent on the parameters of the controller. Thus, how to quickly obtain the optimal system parameters for better controller performance becomes a big challenge. According to Eqs (15–20), the derivative of NFTSMC sliding mode surface can be derived as follows:


$$\begin{array}{cc} & \dot{S}=\dot{e}+k_{1}\lambda |e{|}^{\lambda -1}\cdot \dot{e}+k_{2}\frac{p}{q}|\dot{e}{|}^{(p/q)-1}\cdot \ddot{e}\\ & =\dot{e}+k_{1}\dot{e}+k_{1}\lambda |e{|}^{\lambda -1}\cdot \dot{e}+k_{2}\frac{p}{q}|\dot{e}{|}^{(p/q)-1}.({\dot{x}}_{2}-{\ddot{x}}_{d})\\ & =\dot{e}+k_{1}\dot{e}+k\lambda |e{|}^{\lambda -1}\cdot \dot{e}+k_{2}\frac{p}{q}|\dot{e}{|}^{(p/q)-1}\cdot (M^{-1}(x_{1})u-M^{-1}C(x_{1},x_{2})x_{2}-\Delta d-{\ddot{x}}_{d}) \end{array}$$ (21)

Thus, the PID sliding mode surface can be calculated as follows:


$$\begin{array}{cc} & S_{\text{PID}}=K_{P}s+K_{I}\int_{0}^{t}sdt+K_{D}(\dot{e}+k_{1}\dot{e}+k\lambda |e{|}^{\lambda -1}\cdot \dot{e}\\ & +k_{2}\frac{p}{q}|\dot{e}{|}^{(p/q)-1}\cdot (M^{-1}(x_{1})u-M^{-1}C(x_{1},x_{2})x_{2}-\Delta d-{\ddot{x}}_{d})) \end{array}$$ (22)

where

$\Xi (x_{1},x_{2},x_{d},{\dot{x}}_{d},{\ddot{x}}_{d})=K_{P}s+K_{I}\int_{0}^{t}sdt+K_{D}(\dot{e}+k_{1}\dot{e}+k\lambda |e{|}^{\lambda -1}\cdot \dot{e}-k_{2}\frac{p}{q}|\dot{e}{|}^{(p/q)-1}\cdot (M^{-1}C(x_{1},x_{2})x_{2}+{\ddot{x}}_{d}))$ $\Lambda (\dot{e},x_{1})=K_{D}k_{2}\frac{p}{q}|\dot{e}{|}^{(p/q)-1}\cdot M^{-1}(x_{1})$ and $\Theta (\dot{e},\Delta d)=K_{D}k_{2}\frac{p}{q}|\dot{e}{|}^{(p/q)-1}\cdot \Delta d$.

Thus, Eq (22) can be simplified as follows:


$$S_{\text{PID}}=\Xi (x_{1},x_{2},x_{d},{\dot{x}}_{d},{\ddot{x}}_{d})+\Lambda (\dot{e},x_{1})+\Theta (\dot{e},\Delta d)$$ (23)

Therefore, the PID-NFTSMC control law u can be designed as follows:


$$u=-\Lambda {\left(\dot{e},x_{1}\right)}^{-1}(u_{1}+u_{2})$$ (24)

where $\Lambda {\left(\dot{e},x_{1}\right)}^{-1}$ denotes the pseudo-inverse of $\Lambda (\dot{e},x_{1})$. The equivalent control can be designed as follows:


$$u_{1}=\Xi (x_{1},x_{2},x_{d},{\dot{x}}_{d},{\ddot{x}}_{d})$$ (25)

And an integral of a switching term is defined as follows:


$${\dot{u}}_{2}=(\beta +\eta )sign(S_{PID})$$ (26)

The design of output law can eliminate chattering behavior when reaching the sliding mode surface. Where $|\beta |\leq \frac{d\Theta (\dot{e},\Delta d)}{dt}$ and η is a small positive constant.

**Theorem 1.** Consider the system model described in Eq. (14) and the proposed PID-NFTSMC sliding surface in Eqs. (19 and 20). If the composite controller in Eqs. (24–26) are employed as the control input to the system, then the sliding surface is stable and convergent to zero.

**Proof.** Inserting Eqs. (24–26) into Eq. (23), we can obtain


$$S_{PID}=-u_{2}+\Theta (\dot{e},\Delta d)$$ (27)

Differentiating the sliding variable in Eq. (27) with respect to time, we can have


$${\dot{S}}_{\text{PID}}=-{\dot{u}}_{2}+\Theta (\dot{e},\Delta d)/dt$$ (28)

Consider the following Lyapunov function candidate:


$$V=\frac{1}{2}S_{\text{PID}}^{T}S_{\text{PID}}$$ (29)

Based on Eqs (21–26), the derivation of Lyapunov function can be calculated as follows:


$$\begin{array}{cc} & \dot{V}=S_{\text{PID}}^{T}{\dot{S}}_{\text{PID}}\\ & =S_{\text{PID}}^{T}(-{\dot{u}}_{2}+\Theta (\dot{e},\Delta d)/dt)\\ & =S_{\text{PID}}^{T}(-(\beta +\eta )sign(S_{PID})+\Theta (\dot{e},\Delta d)/dt)\\ & =-\beta |S_{PID}|+S_{PID}\Theta (\dot{e},\Delta d)/dt-\eta |S_{PID}|\\ & \leq -\eta |S_{PID}| \end{array}$$ (30)

Consequently, the PID-NFTSCM sliding surface is stable and convergent based on the results of Lyapunov criterion.

**Remark 1.** Compared with other traditional sliding mode surfaces, the proposed PID sliding surface has three main terms. First, the K_p_s(t) is utilized to maintain the properties of the traditional NFTSMC. Second, $K_{I}\int_{0}^{t}s(t)$ helps to get high robustness property that is similar to a manner to the integral SMC. Third, the introduction of $K_{D}\frac{ds(t)}{dt}$ can help to eliminate chattering of high order sliding mode controller. Thus, the proposed PID-NFTSMC can have major benefits of the NFTSMC, the integral SMC and high order sliding mode controller simultaneously.

### 3.2. Crested porcupine optimizer (CPO) for parameter tuning

Algorithm 1

Pseudo code of CPO.

***Start***

  Set parameters $N,T_{max},\partial ,T_{f},T,N_{min}$;

  Initialize the solutions’ positions randomly $X^{i},i=1,2,...,N$;

***While*** ($t<T_{\max}$)

  Evaluate fitness values for the candidate solutions;

  Determine the best solution so far;

  Update γ_t_ using Eq. (31);

  Update the population size N using Eq. (32);

  ***For*** i = 1: N ***do***

    Generate two random numbers, $\tau_{8},\tau_{9}$

    **If** $\tau_{8}<\tau_{9}$ %% ***Exploration phase***

    Generate two random numbers, $\tau_{6},\tau_{7}$

    **If** $\tau_{6}<\tau_{7}$ %% First defense mechanism

    **Else** % % Second defense mechanism

    **Else *%% Exploitation phase***

    Generate two random numbers, $\tau_{10}$

    **If** $\tau_{10}<T_{f}$ %% Third defense mechanism

    **Else %%** Fourth defense mechanism

    **End if**

    **If** $f(\vec{x_{i}^{t+1}})>f(\vec{x_{i}^{t}})$

    $\vec{x_{i}^{t+1}}=\vec{x_{i}^{t}}$

    **End if**

    t=t+1

   **End for**

  ***End while***

  ***Return***

 **Stop**

To achieve a fast response with minimal trajectory tracking errors, the parameters of the PID and NFTSMC controllers were optimized using the Crested Porcupine Optimization (CPO) algorithm. This algorithm, introduced in 2023, draws inspiration from the natural defensive mechanisms of the crested porcupine. It translates the porcupine's multi-stage defense strategies into a structured exploration and exploitation framework for optimization, enabling the effective search for globally optimal solutions within the parameter space. The crested porcupine's defense mechanisms are initiated with visual and auditory cues in response to minor threats. However, when facing more severe threats, these responses are strengthened by olfactory signals and physical attacks. CPO algorithmically models these four defense strategies into a progressive optimization process, transitioning from a broad global search phase (analogous to visual and auditory defenses) to a focused local exploitation phase (comparable to olfactory and physical responses). By integrating these complementary strategies, CPO ensures a comprehensive exploration of the search space while refining solutions in promising regions.

**Fig 7:**
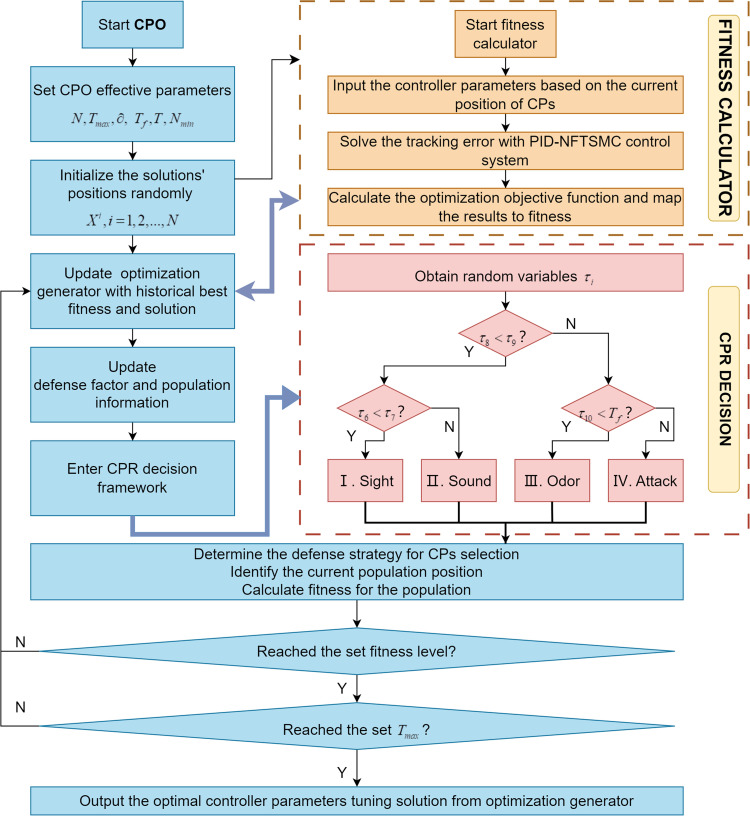
Optimization flowchart of PID-NFTSMC parameters with CPO.

As shown in Fig 7, the key parameters of CPO algorithm were set firstly including the initial population size N, the maximum number of function evaluations $T_{\max}$, the convergence speed factor ∂, a predefined constant that balances local and global exploitation phases $T_{f}\in (0,1)$, a variable determining the number of population reduction cycles T and the minimum permissible population size $N_{\min}$. Then, a set of candidate solutions are generated randomly.

Secondly, when $t<T_{\max}$, the fitness values of the candidate solutions were calculated to determine the optimal solution. Meanwhile, the defense factor $\gamma_{t}$ needs to be updated:


$$\gamma_{t}=2\times \text{rand}\times {\left(1-\frac{t}{t_{\max}}\right)}^{\frac{t}{t_{\max}}}$$ (31)

In order to accelerate the convergence of the algorithm while maintaining population diversity, CPO introduces a cyclic population reduction technique. Based on Eq (30), the population size was updated after iteration.


$$N=N\times \text{rand}\times {\left(1-\frac{t}{t_{\max}}\right)}^{\frac{t}{t_{\max}}}$$ (32)

where % is the remainder or modulus operator. Thus, this strategy allows the algorithm to focus on superior regions with a smaller population size in the later iterations, improving computational efficiency and accelerating convergence to the global optimum.

Thirdly, in each iteration, the CPO algorithm determines whether the current stage is exploratory or exploitative by comparing random parameters ($\tau_{6},\tau_{7},\tau_{8},\tau_{9},\tau_{10}$) and control parameters $T_{f}$. When $\tau_{8}<\tau_{9}$, the algorithm enters the exploration phase. When $\tau_{6}<\tau_{7}$, it engages the first defense mechanism. Otherwise, it engages the second mechanism. This phase is designed to search globally and enhance the ability to jump out of local extremes. When $\tau_{8}>\tau_{9}$, it enters the exploration phase responding to more intense sniffing and physical attack strategies. When $\tau_{10}<T_{f}$, it engages the third defense mechanism. Otherwise, it enters the fourth defense mechanism. Thus, this multilevel defense strategy enables the CPO to flexibly schedule the global and local search intensity during the iteration process, effectively avoiding early stagnation in the region of poor-quality solutions.

Lastly, the CPO algorithm updates and iterates until $t=T_{\max}$. The optimal solution obtained by CPO algorithm is a set of PID and NFTSMC controller parameters that are approximately globally optimal. Therefore, it reduces the time for tuning parameters by manual and provides superior performance and robustness.

### 3.3. Fitness function and parameter configuration

To achieve superior dynamics performances and reduce the times of tuning parameters, the main parameters of PID-NFTSMC controllers ($K_{P},K_{I},K_{D},k_{1},k_{2},\beta$) were optimized through CPO algorithm. These main controller parameters make up a six-dimension vector $X_{\max}=K_{P},K_{I},K_{D},k_{1},k_{2},\beta$. The trajectory tracking error of three joints represented as $e_{j1},e_{j2},e_{j3}$ respectively. Because the main objective of the proposed method was to obtain high accuracy trajectory tracking, the fitness function of CPO algorithm can be described as Mean Absolute Error (MAE):


$$MAE=\frac{1}{N}\sum_{i=1}^{N}|e_{j1}|+|e_{j2}|+|e_{j3}|$$ (33)

## 4. Results and discussion

### 4.1. Simulation setup

**Fig 8:**
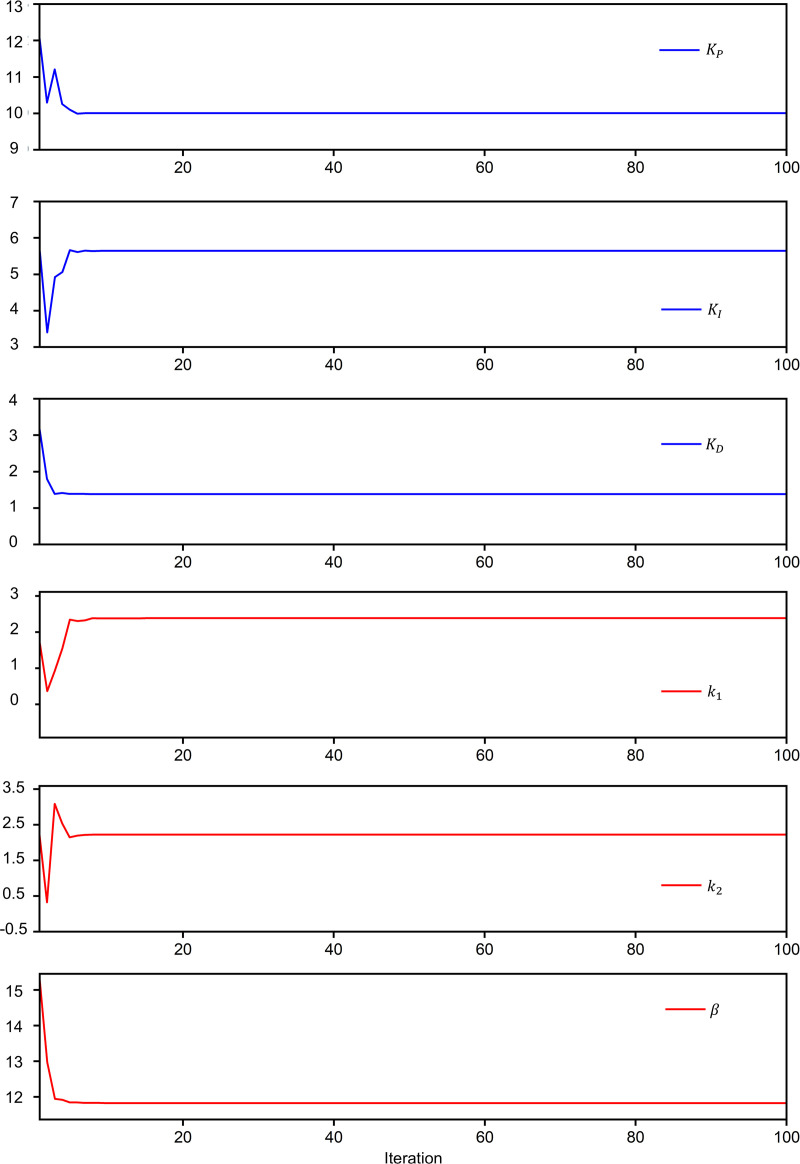
Evolutions of self-tuning controller parameters.

**Fig 9:**
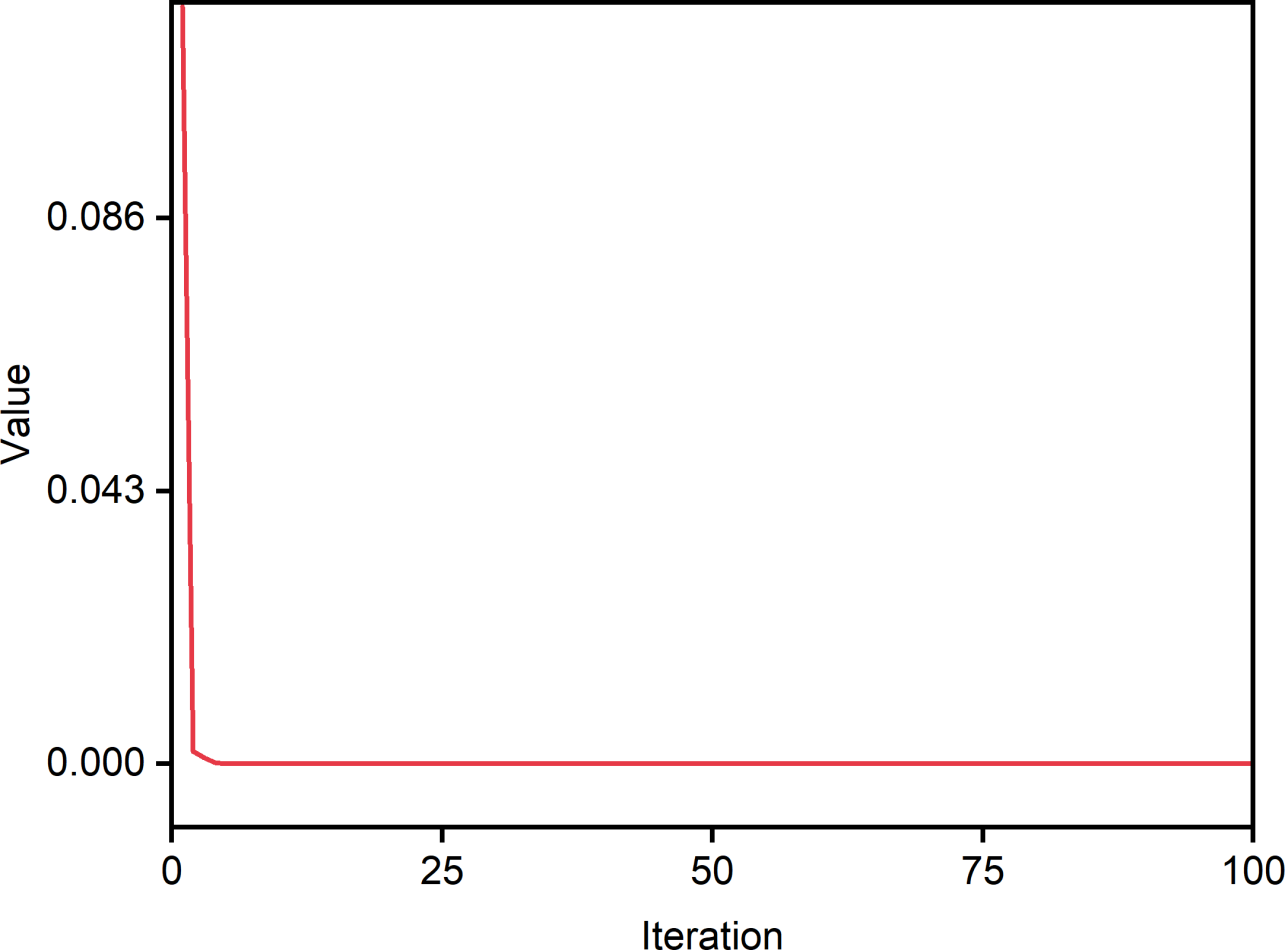
Convergence curve of CPO.


In this study, to further verify the performance of the proposed self-tuning PID-NFTSMC controller, multi-comparison simulations were utilized. The simulation results of the proposed controller were compared with the PID controller, SMC controller, NFTSMC controller and PID-SMC controller. As shown in Table 4, the relevant controller parameters of these controllers were achieved using trial and error method until optimal parameters were obtained. The main parameters of PID-NFTSMC controllers were obtained by CPO optimization algorithm. In CPO optimization algorithm, *N* = 100, *T* = 100, *T*_*f*_* = *10, *N*_*min*_* =* 10. The boundary constraints on the controller parameters were defined as $X_{\max}=[15,10,5,5,5,30]$ and $X_{\min}=[0.1,0.1,0.1,0.1,0.1,1]$. As shown in Fig 8, after 100 iterations, $K_{P},K_{I},K_{D},k_{1},k_{2},\beta$ converge gradually. Also, as shown in Fig 9, the convergence value decreased gradually to a small value after several iterations. There results show that the CPO algorithm can find an approximate global optimal solution successfully with fast speed. Finally, these optimal controller parameters were 10, 5, 1, 2, 3, 15 respectively. The corresponding switching control parameters for the other controllers (SMC, NFTSMC, PID-SMC) were then adjusted to align with those of the PID-NFTSMC controller. This approach ensured that all the controllers were based on the same switching control principles [40]. In addition, the external disturbances and uncertainty in the concrete pouring construction robots were assumed as follows:

**Table 4:** Main parameters of the investigated controller.

Controller	Parameters	Value
PID	$K_{P},K_{I},K_{D}$	600000, 45550, 73550
SMC	λ,β,η	2, 15, 2
NFTSMC	$k_{1},k_{2},p,q,\lambda ,\beta ,\eta$	2, 3, 6, 5, 2, 15, 2
PID-SMC	$K_{P},K_{I},K_{D},\lambda ,\beta ,\eta$	10, 5, 1, 2, 15, 2
PID-NFTSMC	$K_{P},K_{I},K_{D},k_{1},k_{2},p,q,\lambda ,\beta ,\eta$	10, 5, 1, 2, 3, 6, 5, 2, 15, 2

$$\Delta d=\left[\begin{array}{cc} & 2.3\;x_{2}-1.2\;\sin(2x_{2})+0.95\;\sin(x_{2})\\ & 2.3x_{2}-1.2sin(2x_{2})+0.95sin(x_{2})\\ & -2.1x_{2}-1.6sin(3x_{2})+0.75sin(x_{2}) \end{array}\right]$$ (34)

The desired trajectory of the proposed concrete pouring construction robots was defined as follows:


$$x_{d}=\left[\begin{array}{cc} & cos(2t/\pi )-1\\ & cos(2t/\pi +2\pi )\\ & sin(2t/\pi +2\pi )-1 \end{array}\right]$$ (35)

### 4.2. Tracking accuracy comparison

**Fig 10:**
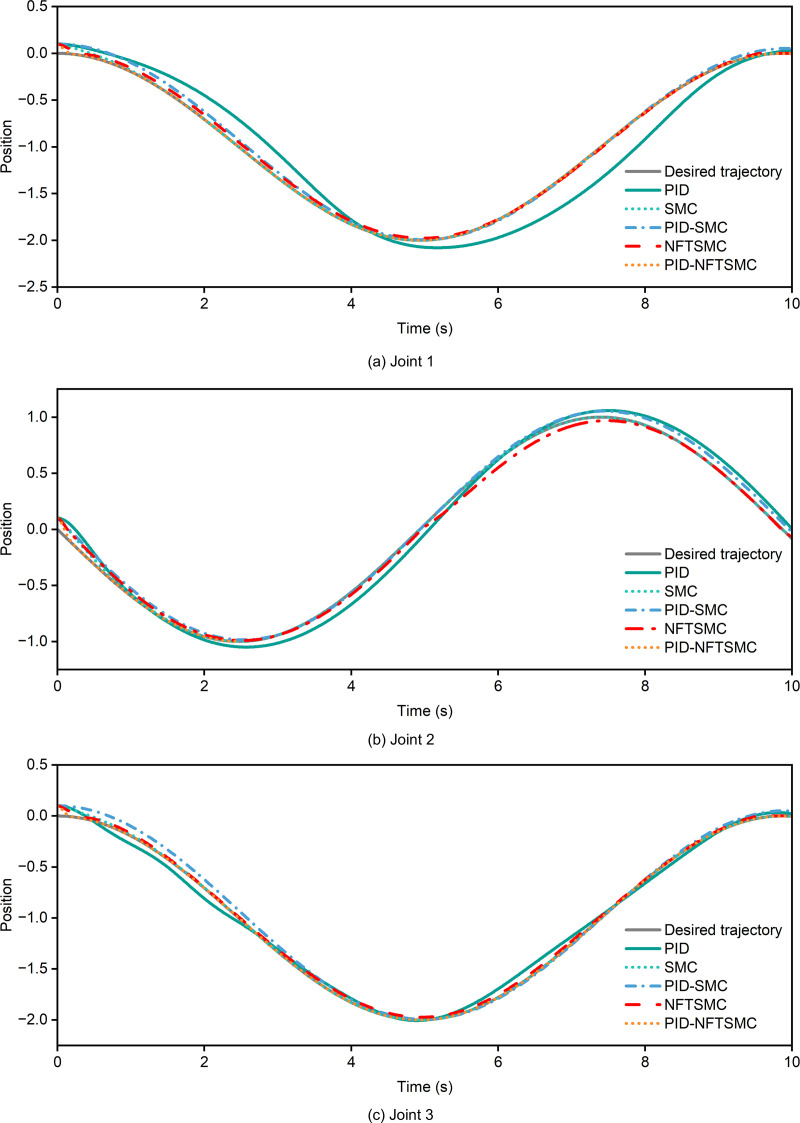
Tracking of desired rajectory. (a) joint 1 (b) joint 2 (3) joint 3.

**Fig 11:**
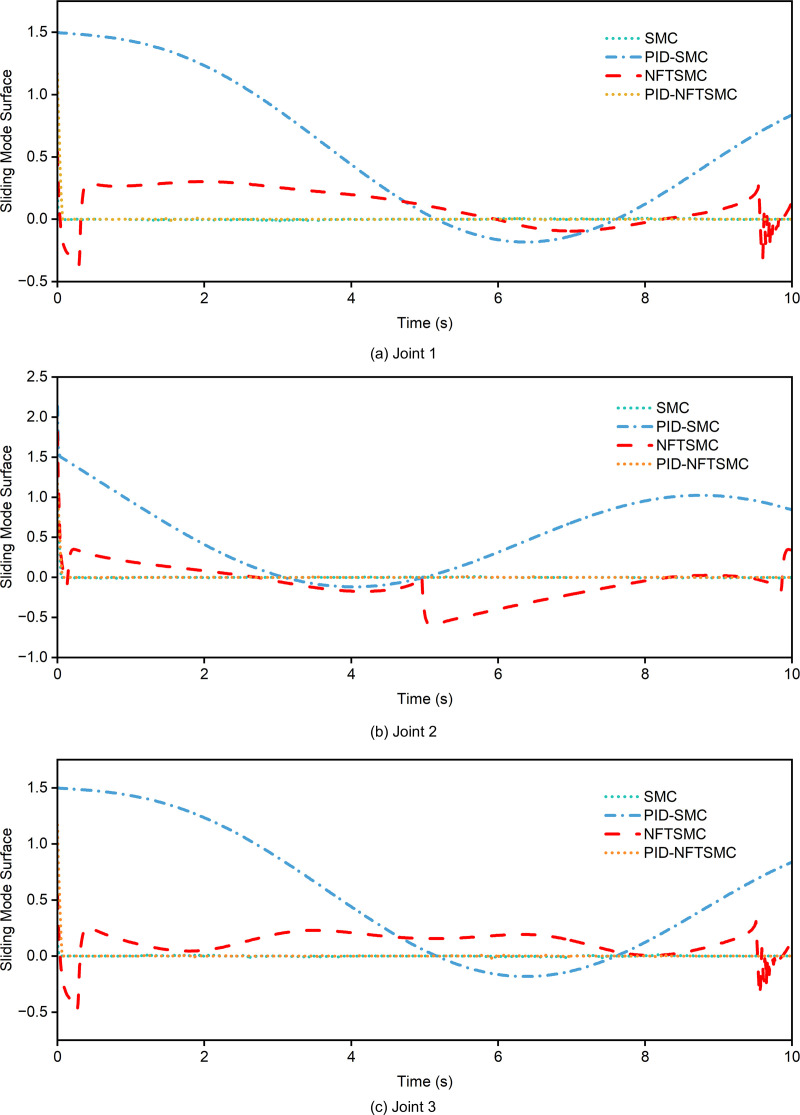
Comparison of sliding mode surface for different control methods. (a) joint 1 (b) joint 2 (3) joint 3.

The tracking performance of various controllers for the desired trajectory is illustrated in Fig 10. The gray solid line represents the desired trajectory of the three joints. As shown in Fig 10a, the proposed PID-NFTSMC controller demonstrates superior dynamic performance and stability for Joint 1. The trajectory of joint 1 closely aligns with the desired trajectory, achieving nearly perfect tracking with high accuracy and a rapid response. In contrast, the PID-SMC controller (depicted by the blue dash-dot line) shows minor deviations, particularly between 4–6 seconds and around 8 seconds. The SMC controller (cyan dotted line) and the conventional PID controller (green solid line) exhibit more pronounced deviations during rapid trajectory changes, such as those occurring between 4–6 seconds and 7–9 seconds. Notably, the PID-NFTSMC controller achieves the fastest response to dynamic changes without significant overshoot, whereas the other controllers show minor offsets, indicating less effective handling of nonlinear uncertainties (as shown in Fig 11). For Joint 2, the PID-NFTSMC controller maintains its superior performance, showing minimal deviations across the trajectory. However, the NFTSMC controller exhibits noticeable errors between 6–8 seconds, while the PID and SMC controllers display even larger deviations, particularly during rapid trajectory changes. For Joint 3, the errors of all controllers are relatively smaller compared to Joints 1 and 2. This reduction in error may be attributed to the simpler dynamics associated with Joint 3. Among all controllers, the PID-NFTSMC controller remains optimal, with negligible tracking errors.

**Fig 12:**
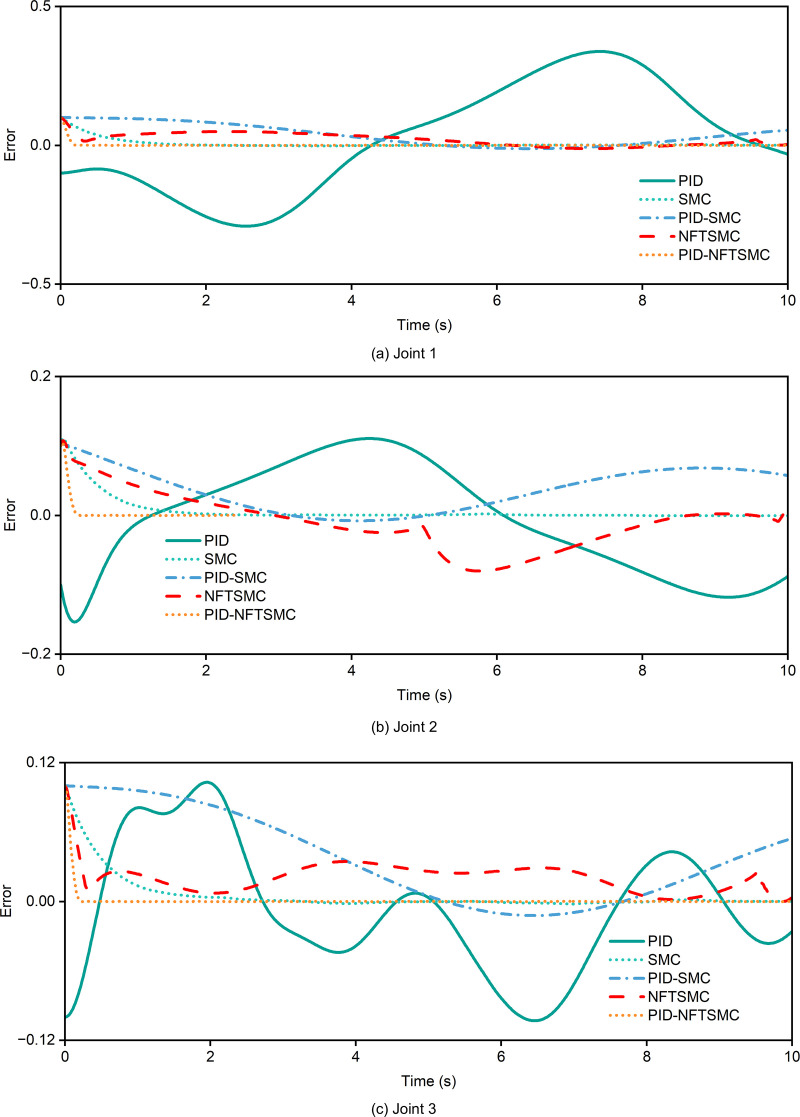
Trajectory tracking error. (a) joint 1 (b) joint 2 (3) joint 3.

The trajectory tracking error of each controller is shown in Fig 12. As depicted in Fig 12a, the tracking error for Joint 1 using the PID-NFTSMC controller is nearly zero across the entire time range, with minimal overshoot or deviation, even in dynamically changing regions (e.g., 4–6 seconds). According to Table 5, the maximum tracking error of the PID-NFTSMC controller is 0.098740, and the root-mean-square (RMS) error is 0.007405. These results highlight the controller's exceptional accuracy and dynamic response capabilities. In comparison, the PID controller exhibits a significantly higher maximum error of 0.138130 and an RMS error of 0.196937. Moreover, the PID controller struggles with large fluctuations during rapid trajectory changes, such as those occurring from 4–6 seconds, indicating its limited ability to handle dynamic conditions effectively. The SMC and PID-SMC controllers achieve moderate tracking errors, with the PID-SMC controller performing slightly better but still falling short of the stability achieved by the PID-NFTSMC controller. Meanwhile, the NFTSMC controller experiences larger and more fluctuating errors, especially in regions with rapid dynamic changes, further underscoring the superior performance of the PID-NFTSMC controller in dynamic trajectory tracking scenarios.

**Table 5:** Comparison of maximum and rms value of tracking error.

Controller	$E_{\max\_1}$	$E_{rms\_1}$	$E_{\max\_2}$	$E_{rms\_2}$	$E_{\max\_3}$	$E_{rms\_3}$
PID	0.138130	0.196937	0.110720	0.079091	0.103060	0.055350
SMC	0.100000	0.011289	0.110090	0.007977	0.099760	0.011398
NFTSMC	0.100000	0.001570	0.107170	0.042660	0.100000	0.001690
PID-SMC	0.100000	0.049240	0.103420	0.046720	0.100000	0.049240
PID-NFTSMC	0.098740	0.007405	0.105880	0.009859	0.088740	0.007605

For Joint 2, the maximum error and root-mean-square (RMS) error of the PID-NFTSMC controller are 0.105880 and 0.009859, respectively. The error remains stable during rapid trajectory changes, particularly between 6 and 8 seconds, without significant deviations, which highlights its superior robustness. In contrast, the NFTSMC controller exhibits a maximum error of 0.107170 and an RMS error of 0.042660. A significant deviation occurs after 6 seconds, likely due to the complex dynamics of Joint 2, where NFTSMC struggles to fully address the system's nonlinearities. The PID and SMC controllers demonstrate poor performance, especially the PID controller, which has a maximum error of 0.110720 and an RMS error of 0.079091, with noticeable fluctuations during dynamic changes. Therefore, for the highly nonlinear dynamics of Joint 2, the PID-NFTSMC controller demonstrates superior performance compared to other strategies. For Joint 3, as shown in Fig 12c and detailed in Table 5, the PID-NFTSMC controller achieves a minimum error of 0.088740 and an RMS error of 0.007605. This represents better dynamic performance and robustness compared to other controllers. The NFTSMC controller, on the other hand, shows a maximum error of 0.100000 and an RMS error of 0.001690, with noticeable fluctuations, especially during dynamic transitions after 6 seconds. The PID controller exhibits even greater fluctuations in these regions. Specifically, the PID controller has a minimum error of 0.103060 and an RMS error of 0.055350, which are lower than the errors observed in Joints 1 and 2 but remain significantly higher than those of the PID-NFTSMC controller.

### 4.3. Convergence time analysis

As shown in Table 6, a comparative analysis of convergence time was performed. Here, convergence time was defined as the time required for the system error to stabilize below 0.01. CT_1_, CT_2_ and CT_3_ represented as convergence time for joint 1, joint 2 and joint 3. Our results indicated that the self-tuning PID-NFTSMC controller exhibited the shortest convergence for all joints (CT₁ = 0.1553s, CT₂ = 0.1540s, and CT₃ = 0.0100s). This performance was significantly superior to other methods, such as PID (>10s), NFTSMC (~8.10s–9.65s), and PID-SMC (>10s). Although the SMC controller (CT₁ = 1.1507s, CT₂ = 1.2256s, CT₃ = 1.1639s) demonstrated better performance than PID-based methods, it could not outperform the self-tuning PID-NFTSMC controller. These results further verified the effectiveness of self-tuning PID-NFTSMC controller in achieving fast response, stability, and robustness against external disturbances.

**Table 6:** Comparison of convergence time (CT) of the system.

Controller	$CT_{1}$	$CT_{2}$	$CT_{3}$
PID	>10	>10	>10
SMC	1.1507	1.2256	1.1639
NFTSMC	9.6487	8.1010	9.6503
PID-SMC	>10	>10	>10
PID-NFTSMC	0.1553	0.1540	0.0100

### 4.4. Power, ISV and ITSV analysis

**Fig 13:**
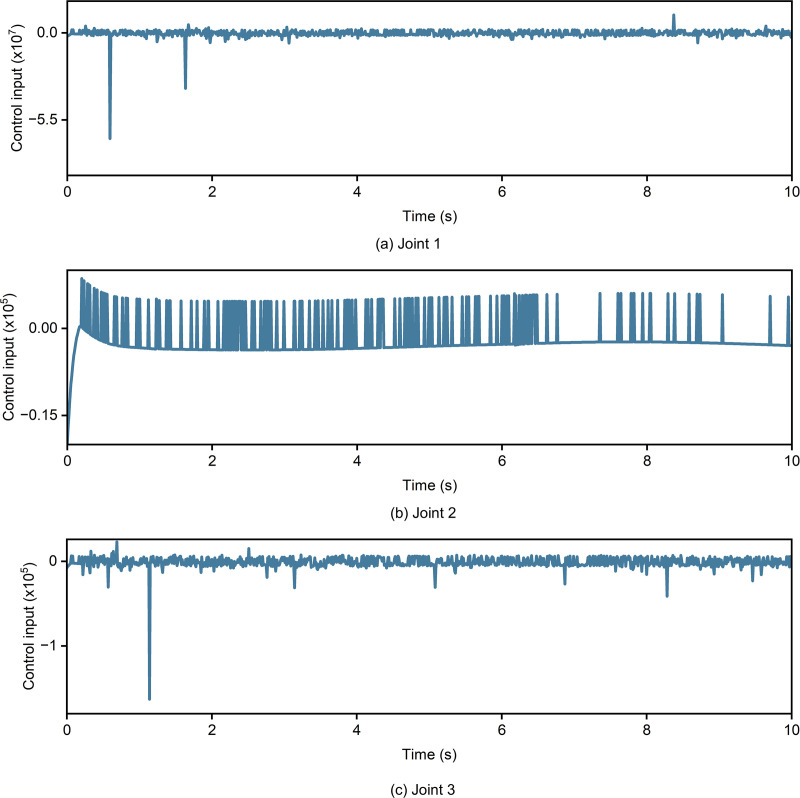
Control input. (a) joint 1 (b) joint 2 (3) joint 3.

In order to evaluate the overall performance of different controllers, three main indicators were introduced, including power consumption, input signal variation (ISV) and integrated time-weighted signal variation (ITSV). As shown in Table 7, compared to the traditional NFTSMC, PID-NFTSMC indicated slightly higher energy consumption but only improved the control performance metrics by about 10 times. In addition, three different joints consumed significantly less energy than conventional SMC. However, compared with PID, its energy consumption was slightly higher but PID-NFTSMC showed superior dynamics performance and fast convergence time. Compared with traditional NFTSMC and SMC, PID-NFTSMC significantly reduced ISV and effectively suppressed chattering. Although it was not as stable as PID-SMC, it maintained reasonable stableness under nonlinear disturbance conditions. Thus, it was better suited to complex environments. Compared with traditional PID, PID-SMC and NFTSMC controller, the ISTV of PID-NFTSMC was obviously higher. But it improved stability by three orders of magnitude compared to traditional SMC controller. As shown in Fig 13, while ensuring robustness against external disturbances and variations of system parameters, its later control signal tends to be stable without continuous chattering.

**Table 7:** Comparison of power consumption, ISV and ITSV of different controllers.

Controller	Power Consumption			ISV			ITSV		
	Joint 1	Joint 2	Joint 3	Joint 1	Joint 2	Joint 3	Joint 1	Joint 2	Joint 3
PID	5.26 × 10⁵	1.74 × 10⁵	1.31 × 10⁵	1.29 × 10⁴	3.56 × 10³	6.83 × 10³	3.97 × 10⁶	1.33 × 10⁶	2.29 × 10⁶
SMC	5.62 × 10⁶	1.49 × 10⁶	1.92 × 10⁶	2.49 × 10⁶	6.13 × 10⁵	7.95 × 10⁵	4.02 × 10^12^	7.96 × 10¹¹	1.31 × 10¹²
NFTSMC	4.50 × 10⁵	1.20 × 10⁵	1.11 × 10⁵	1.88 × 10⁶	1.10 × 10⁵	7.38 × 10⁵	8.97 × 10⁷	2.14 × 10⁶	3.52 × 10⁷
PID-SMC	6.39 × 10⁵	6.59 × 10⁴	2.52 × 10⁵	8.54 × 10²	2.44 × 10³	3.04 × 10²	3.80 × 10⁶	5.02 × 10⁵	1.67 × 10⁶
PID-NFTSMC	4.99 × 10⁶	1.40 × 10⁶	1.73 × 10⁶	1.59 × 10⁶	4.40 × 10⁵	5.14 × 10⁵	7.78 × 10⁹	2.21 × 10⁹	2.69 × 10⁹

Overall, the self-tuning PID-NFTSMC controller outperforms other controllers across all joints, particularly in terms of tracking accuracy and robustness. Meanwhile, compared with other traditional methods, the proposed method significantly reduced convergence time. Thus, a concrete pouring construction robot can obtain fast and precise trajectory tracking against external disturbances. Additionally, as depicted in Fig 13, the output control signals for the three joints using the PID-NFTSMC controller show significantly reduced chattering, further validating the effectiveness of the proposed control method.

## 5. Conclusion

In this study, a PID-NFTSMC controller was redesigned and applied to a concrete pouring construction robot based on its dynamic model. To further enhance dynamic performance, a Crested Porcupine Optimization algorithm was developed, resulting in a self-tuning PID-NFTSMC control method. Compared to conventional controllers, the proposed approach significantly improves dynamic response and reduces overshoot. The integration of PID and NFTSMC control strategies enables the robot to achieve high-precision trajectory tracking with minimal steady-state errors, even in the presence of external disturbances and system uncertainties. Additionally, the automatic parameter tuning of the PID and NFTSMC components reduces the computational burden, thereby improving the overall robustness and reliability of the system. The designed control law also effectively eliminates chattering, ensuring smoother operation. However, the applicability and stability of this control method under real-world complex conditions have not yet been thoroughly investigated. Future research could focus on validating the controller in practical scenarios, such as multi-tasking environments, to assess its performance and reliability in real-world applications.
